# Transcatheter mitral valve replacement in failed surgical annuloplasty ring with Impella support

**DOI:** 10.1007/s12928-024-01068-4

**Published:** 2024-12-06

**Authors:** Daisuke Hachinohe, Ryo Horita, Shah Sagar, Ryo Ohtake, Hidemasa Shitan, Kazuki Mizutani

**Affiliations:** 1 Cardiovascular Medicine, Sapporo Heart Center, Sapporo Cardio Vascular Clinic, 8-1, Kita-49 Higashi-16, Higashiku, Sapporo, 007-0849 Japan; 2 Baroda Heart Institute and Research Centre, Vadodara, India

**Keywords:** Edwards SAPIEN 3 Ultra RESILIA, Failed ring, TMVI, Transcatheter mitral valve interventions, Valve-in-ring, IMPELLA

A 59-year-old male with a history of CABG and mitral annuloplasty using a 30 mm Physio II ring (Edwards Lifesciences, Irvine, California) 9 years ago was transferred to our institution due to congestive heart failure. Transthoracic echocardiography (TTE) revealed severe mitral regurgitation (MR) with a regurgitant volume of 62 mL and an effective regurgitant orifice (ERO) of 0.46 cm^2^ (Fig. 1A, Supplemental movie). Transesophageal echocardiography (TEE) showed significant bileaflet tethering, with a mitral valve (MV) area of 3.7 cm^2^ and a mean pressure gradient of 7 mmHg (Fig. 1B–D, Supplemental movie), suggesting a reduced MV area and increased pressure gradient. Despite Impella 5.5 (Abiomed, Danvers, Massachusetts) support, heart failure was attributed to severe MR due to failure of the surgical annuloplasty ring, necessitating further MV intervention. Given the patient's poor overall condition, surgical procedure was deemed high risk. Transcatheter edge-to-edge repair was considered but likely to result in hemodynamic mitral stenosis with an unacceptable residual MR. Therefore, transcatheter MV replacement in a failed ring (TMVR-in-R) was performed using a SAPIEN 3 Ultra RESILIA valve (Edwards Lifesciences), as previously described [1]. This treatment, not approved in Japan, was performed with the approval of our Institutional Review Board. In this case, the preoperative CT indicated a neo-left ventricular outflow tract (LVOT) area of 7.66 cm^2^, suggesting a low risk of post-procedural LVOT obstruction. A 26 mm device, with an oversizing ratio of 27.5%, was deployed under rapid pacing at 180 bpm, with Impella support temporarily stopped to ensure stability (Fig. 1F, Supplemental movie). This approach aimed to enhance anchoring, conformability, and THV apposition to the ring, thereby reducing the risk of paravalvular leak (PVL). Post-procedure, PVL was dramatically reduced, showing only trivial (Fig. 1G, H, Supplemental movie), and the Impella could be removed after 5 days. The patient was discharged on the 28th day after admission.

**Fig. 1:**
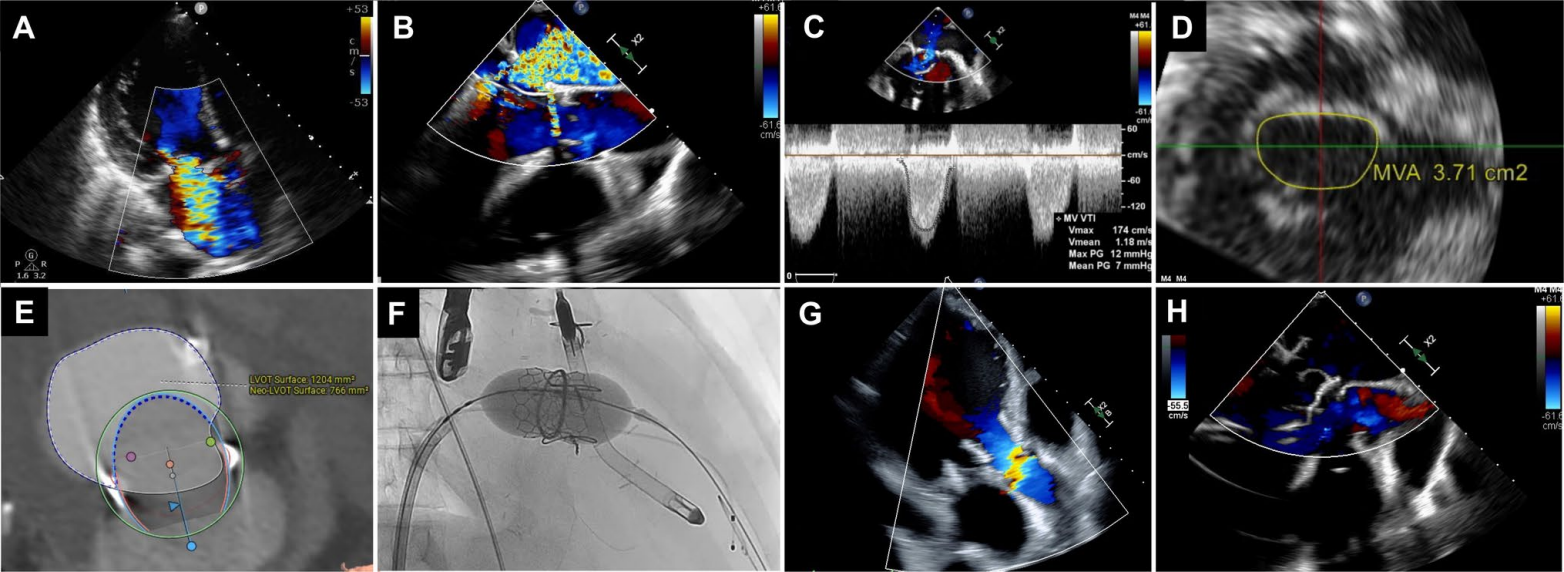
**A** Preoperative transthoracic echocardiography (TTE), **B**–**D** preoperative transesophageal echocardiography (TEE), **E** Preoperative CT demonstrated a neo-left ventricular outflow tract area measuring 7.66 cm^2^, **F** transcatheter mitral valve replacement in a failed ring, **G** postoperative TTE, and **H** postoperative TEE

Although rapid pacing generally stabilizes valve deployment, it may be insufficient under Impella support due to high residual blood pressure, leading to unpredictable valve behavior. Therefore, temporarily discontinuing Impella support during deployment is crucial for control. TMVR-in-R significantly improves severe MR in failed rings, even in cases requiring Impella, highlighting key considerations for safely performing TMVR-in-R with Impella.

## Supplementary Information

Below is the link to the electronic supplementary material.

Supplementary file1 (MP4 23467 KB)

## Data Availability

The data of the current paper is available.
